# Subjective Cognitive Impairment and Mild Cognitive Impairment: risk factors in Mexican community‐dwelling older adults

**DOI:** 10.1002/alz70861_108036

**Published:** 2025-12-23

**Authors:** Neyda Ma. Mendoza Ruvalcaba, Melina Rodríguez Díaz, Karla Patricia Vázquez Núñez

**Affiliations:** ^1^ Universidad de Guadalajara CUTONALA, Tonala, JA Mexico

## Abstract

**Background:**

Subjective cognitive impairment (SCD) and mild cognitive impairment (MCI) are preclinical states in the natural history of Major Neurocognitive Disorder or dementia. SCD has been studied as an indicator of MCI and future dementia, it is defined as persistent self‐experienced decline in cognitive ability despite having normal performance on standardized tests.

**Method:**

Cross‐sectional study, participated n=1190 older adults (from 2018‐2024). Mean age 71.44 years (SD=7.5), education 9.24 years (SD=4.8), 78% women (*n* =927), 53% without a partner. After informed consent, trained gerontologists administered face‐to‐face interviews, measures of global cognitive functioning with the MMSE, meta‐memory, and measurements of risk variables: sociodemographic data, physical and mental health. Data were analyzed with SPSSv28 and Odds Ratios (95% CI) were calculated.

**Result:**

A total of 72.1% of the participants meet the criteria for SCD, and 11.6% for MCI. The significant risk factors for SCD were age (OR=1.31, CI=1.09‐1.72, *p* =.025), sex (OR=2.05, CI=1.54–2.73, *p* <.001), education (OR=1.21, CI=1.03–1.53, *p* =.049), recent life crises (OR=1.55, CI=1.16–2.08, *p* =.002), loss of a loved one (OR=1.32, IC=1.02–1.74, *p* =.023), cancer (OR=2.46CI=1.40–4.34, *p* <.001) and anxiety diagnosis (OR=1.27, CI=1.02–1.71, *p* =.049). The risk factors for MCI were presenting SCD (OR=1.60, CI=1.48–1.74, *p* <.001), having a previous diagnosis of depression (OR=1.54, CI=1.10–2.30, *p* =.016), while having no memory complaints was a protective factor (OR=.622, CI=.432‐.895, *p* =.007).

**Conclusion:**

Detection of subjective cognitive impairment as an early manifestation of cognitive pathology is relevant to prevent or delay progression to MCI and dementia, allowing planning interventions to reverse/reduce the rate of conversion and its consequences on people's lives and public health.

